# Construction and Application of a Private 5G Standalone Medical Network in a Smart Health Environment: Exploratory Practice From China

**DOI:** 10.2196/52404

**Published:** 2024-10-24

**Authors:** Baozhan Chen, Xiaobing Shi, Tianyi Feng, Shuai Jiang, Yunkai Zhai, Mingxing Ren, Dongqing Liu, Chengzeng Wang, Jinghong Gao

**Affiliations:** 1 The First Affiliated Hospital of Zhengzhou University Zhengzhou China; 2 National Engineering Laboratory for Internet Medical Systems and Applications Zhengzhou China; 3 China Academy of Information and Communications Technology Beijing China; 4 Institute for Hospital Management of Henan Province Zhengzhou China; 5 School of Management Engineering Zhengzhou University Zhengzhou China; 6 Henan Institute of Interconnected Intelligent Health Management Zhengzhou China

**Keywords:** 5G, medical private network, construction, application, performance test

## Abstract

**Background:**

To date, the differentiated requirements for network performance in various health care service scenarios—within, outside, and between hospitals—remain a key challenge that restricts the development and implementation of digital medical services.

**Objective:**

This study aims to construct and implement a private 5G (the 5th generation mobile communication technology) standalone (SA) medical network in a smart health environment to meet the diverse needs of various medical services.

**Methods:**

Based on an analysis of network differentiation requirements in medical applications, the system architecture and functional positioning of the proposed private 5G SA medical network are designed and implemented. The system architecture includes the development of exclusive and preferential channels for medical use, as well as an ordinary user channel. A 3-layer network function architecture is designed, encompassing resource, control, and intelligent operation layers to facilitate management arrangements and provide network open services. Core technologies, including edge cloud collaboration; service awareness; and slicing of access, bearer, and core networks, are employed in the construction and application of the 5G SA network.

**Results:**

The construction of the private 5G SA medical network primarily involves system architecture, standards, and security measures. The system, featuring exclusive, preferential, and common channels, supports a variety of medical applications. Relevant standards are adhered to in order to ensure the interaction and sharing of medical service information. Security is achieved through mechanisms such as authentication, abnormal behavior analysis, and dynamic access control. Three typical medical applications that rely on the 5G network in intrahospital, interhospital, and out-of-hospital scenarios—namely, mobile ward rounds, remote first aid, and remote ultrasound—were conducted. Testing of the 5G-enabled mobile ward rounds showed an average download rate of 790 Mbps and an average upload rate of 91 Mbps. Compared with 4G, the 5G network more effectively meets the diverse requirements of various business applications in prehospital emergency scenarios. For remote ultrasound, the average downlink rate of the 5G network is 4.82 Mbps, and the average uplink rate is 2 Mbps, with an average fluctuation of approximately 8 ms. The bandwidth, performance, and delay of the 5G SA network were also examined and confirmed to be effective.

**Conclusions:**

The proposed 5G SA medical network demonstrates strong performance in typical medical applications. Its construction and application could lead to the development of new medical service models and provide valuable references for the further advancement and implementation of 5G networks in other industries, both in China and globally.

## Introduction

Medical institutions are densely populated, with unpredictable business volumes, fluctuating personnel flow, complex demands, and diverse medical service scenarios, all of which pose significant challenges to the network capacity required for their operation and maintenance. In light of the increasingly advanced and rapidly evolving medical services, traditional networking technology is encountering bottlenecks in areas such as the digital transformation of indoor networks, service distribution in transmission networks, intelligent network operations, and end-to-end safety guarantees [1,2]. Particularly during a major public health emergency like the COVID-19 pandemic, when hospitals exceed their normal capacity, health care professionals grappling with this unprecedented crisis face the challenge of effectively coordinating medical resources across widely dispersed locations [3]. Not only have patients been marginalized, but many clinicians have also had limited access to the treatment consultations and guidelines they need from hospitals during the COVID-19 outbreak [4]. As the pandemic continues, relying solely on conventional networking technologies (eg, 3G and 4G) may lead to significant costs and health risks. Studies have emphasized the need to design and implement an indoor network coverage system for hospitals that can meet the multifaceted business requirements and growing traffic demands [3,5]. This system should include features such as capacity redundancy, on-demand configurability, and scalability to ensure adaptability to dynamic changes.

Compared with 4G (the 4th generation mobile communication technology), the emerging 5G (the 5th generation mobile communication technology) mobile communication technology offers higher speeds, lower latency, enhanced capacity, and increased bandwidth. These improvements enable 5G to connect a large number of devices and support massive data processing with greater reliability, making it a game changer in health care services [6,7]. The architecture of the 5G network is divided into non-standalone (NSA) and standalone (SA) configurations [8]. NSA serves as a transitional framework from 4G to 5G, providing 5G mobile bandwidth services by utilizing the existing 4G infrastructure for 5G deployment. It upgrades the current 4G evolved packet core (EPC) network and uses dual connectivity technologies to access 5G New Radio. NSA has become the initial rapid commercial solution for some operators. However, the existing 4G EPC architecture and 4G air interface cannot meet 5G’s requirements for low latency and transmission reliability [9]. SA represents the latest advancements in 5G networks, incorporating state-of-the-art base stations, backhaul infrastructure, and an upgraded EPC. In addition, SA introduces new network elements and interfaces, while adopting advanced technologies such as the Internet of Things (IoT), cloud computing, network virtualization, software-defined networks, and big data [9,10]. Compared with the NSA network architecture, the SA network architecture offers more advantages.

In recent years, advancements in 5G technology have driven the rapid development of health care services. The integration of IoT, big data, and cloud computing has enabled greater interconnectivity among patients, health care professionals, medical devices, and institutions. This has led to the emergence of new eHealth applications, such as mobile health, telemedicine, and internet-based health care [6,11]. These advancements encompass various aspects of disease management, including diagnosis, treatment, rehabilitation, prevention, and health monitoring, effectively enhancing the accessibility and capacity for delivering high-quality medical services [6,12]. However, diverse network performance requirements across different health care service scenarios—both within and between hospitals—continue to pose significant challenges that hinder the advancement of digital and intelligent medical services.

To effectively address the aforementioned challenges and meet the growing demand for network differentiation in medical services, the development of a new 5G-based network is urgently needed. Therefore, this study aims to design and implement a private 5G SA medical network within a smart health environment to accommodate diverse medical service scenarios. First, based on a requirements analysis, this study introduces the overall architecture, functional design, core technologies, and security mechanisms involved in the design and construction of the 5G SA network. Second, the construction and functionality of the 5G network are implemented to achieve the objectives outlined in the requirements analysis. Finally, the performance of the proposed 5G SA network is evaluated through an investigation of 3 representative applications. Lastly, the findings of this study undergo rigorous discussion, leading to the derived conclusions. To the best of our knowledge, this is the first comprehensive account of constructing and applying a private 5G SA medical network within a smart health environment in China. The findings may not only contribute to the further development and implementation of 5G medical networks in China but also provide valuable insights for other regions worldwide.

## Methods

### Requirements Analysis

The 5G network equipment generates a significant volume of diverse data across multiple layers, including the physical, virtual, link, network, and user layers. The current centralized data processing model struggles to keep pace with the low latency and high bandwidth demands of computing loads. Additionally, the traditional network operation and maintenance approach involves numerous manual tasks, which have proven inadequate for meeting the requirements of comprehensive cloud and intelligent network deployment for 5G.

Given the demand for network differentiation in medical applications, the following challenges and requirements must be addressed to construct and implement a private 5G SA medical network within a smart health environment. First, the design of the 5G network should comprehensively consider network coverage, capacity, and cost, taking into account the complexity of medical applications and the variations in transmission loss across different frequency bands and spectrum bandwidths. Second, when building a 5G network, health care facilities should carefully consider the diverse types of medical network application services, as well as the associated network requirements and standards. Third, health care facilities face significant challenges in effectively managing the privacy of information derived from medical devices, health care information systems, and medical networks [13]. Lastly, the concurrent development of a visualization platform for the intelligent operation and maintenance of the 5G network is typically required.

### System Design

#### Overview

The private 5G medical network focuses on 3 major service scenarios: medical monitoring and nursing applications that utilize wireless data collection from hospital medical equipment; medical diagnosis and guidance applications based on video and medical data interaction; and remote-control applications for intelligent medical equipment [5,14]. Based on the specific needs of medical institutions—such as intrahospital mobile medical services, interhospital telemedicine, and out-of-hospital internet medical services—network slicing techniques, mobile edge computing, big data, and artificial intelligence (AI) are used to design and deploy the 5G SA medical industry–specific network. The 5G SA network can meet the diverse security requirements of users in 5G medical services by enabling functions such as network security isolation between the medical private network and public users, flexible deployment of network slices, and dynamic network awareness.

**Figure 1:**
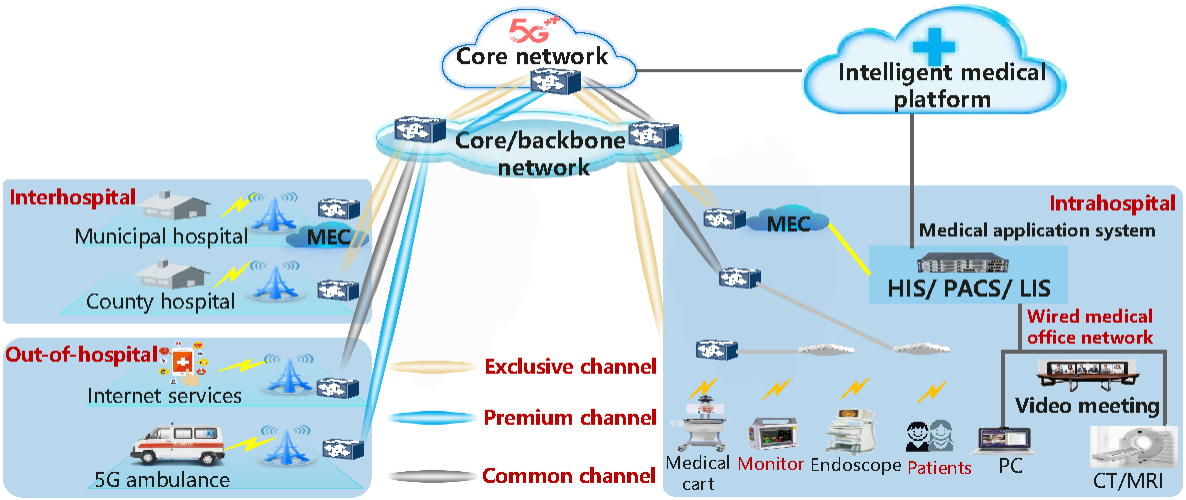
Overall architecture of the proposed private 5G SA medical network. 5G: 5th generation mobile communication technology; CT: computed tomography; HIS: hospital information system; LIS: laboratory information system; MEC: mobile edge computing; MRI: magnetic resonance imaging; PACS: picture archiving and communication system; PC: personal computer; SA: standalone.

Following the service-based architecture of the 5G Core (5GC) network as defined by the 3rd Generation Partnership Project (3GPP) standard and leveraging cloud-native technology, the 5G medical network divides the application scenarios of medical services into network slices. It designs their functions and deploys them at different levels based on the service-based microservice architecture [15]. The private network is constructed by defining the combination of network functional components to cater to diverse medical application scenarios [16]. This approach offers enhanced ubiquitous access, increased flexibility in control and forwarding, and greater network openness for medical institutions [15,17]. Considering the various scenarios of intrahospital, interhospital, and out-of-hospital medical services, the 5G network can be categorized into medical-exclusive and preferential channels, as well as ordinary user channels (Figure 1).

#### Exclusive Channel

The Flexible Ethernet (FlexE) slicing technology is used to partition dedicated network resources for transmitting fixed services within the hospital and collaborative services between hospitals, such as mobile ward rounds, appointment registration, and teleconsultation. This approach ensures guaranteed capabilities regarding delay, speed, reliability, and connection capacity [18]. Additionally, historical data on concurrent business and traffic statistics are combined to plan network resources, which in turn optimizes the allocation of these resources.

#### Premium Channel

It dynamically creates slices to support temporary medical services based on business needs, such as remote first aid, online consultation, and chronic disease management. This approach ensures a seamless experience for temporary medical services and facilitates the support of multiple nested subslices within a main slice for prioritized allocation and meticulous management. Additionally, it strictly isolates traffic between subslices to prevent any interference. It will not disrupt the operational sequence of other services due to sudden traffic. Furthermore, the subslice can be deleted upon completion of the business process, freeing up bandwidth resources, reducing network bandwidth consumption, and achieving cost savings.

#### Common Channel

It primarily supports public user services, allowing patients to access the internet and obtain public services through their mobile phones, tablets, and other mobile devices. These public user services are segregated from both the exclusive and preferential channels to meet the internet needs of patients within the hospital while facilitating isolated operations for medical services across other channels.

### Function Design

#### Network Architecture

**Figure 2:**
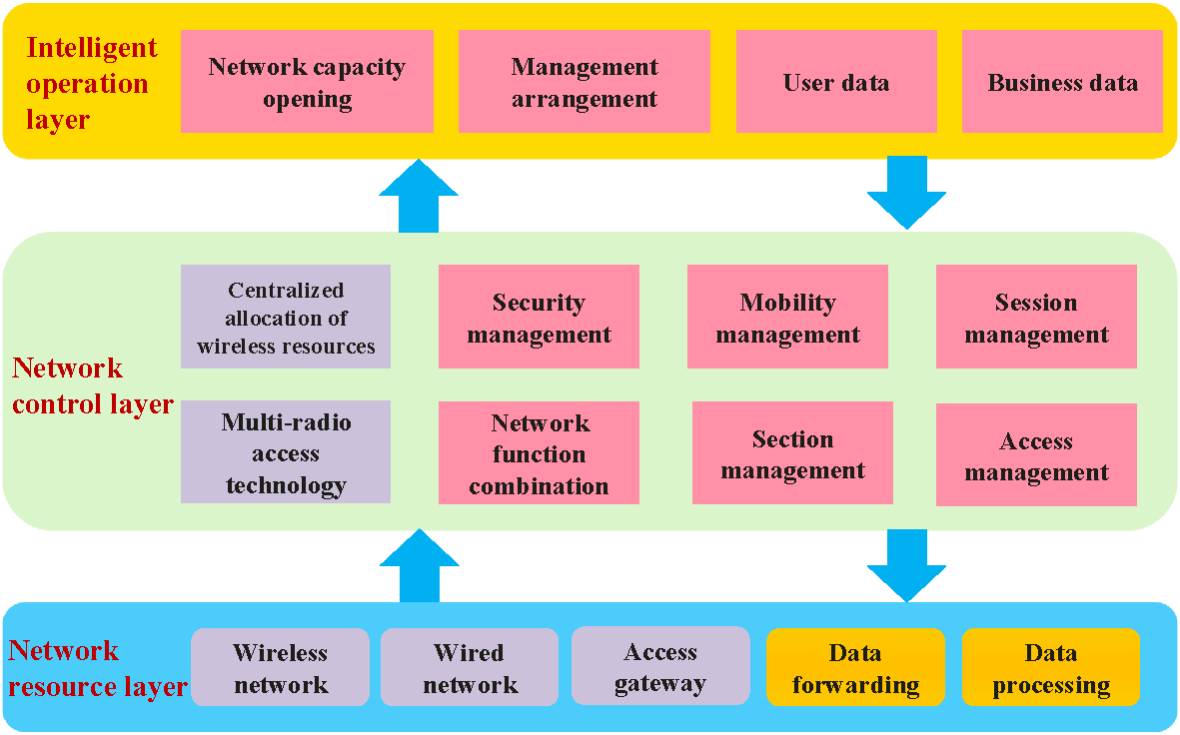
The functional architecture of the proposed private 5G SA medical network. 5G: 5th generation mobile communication technology; SA: standalone.

The proposed private 5G SA network, centered around intelligent operation, is capable of providing resource support for network access and data forwarding functions [19]. It offers an upward management arrangement and open network services, establishing a 3-layer network function architecture (Figure 2).

#### Network Resource Layer

This layer includes functions on both the access and network sides. The access side comprises a central unit and a distribution unit. The central unit primarily provides service aggregation functions, while the distribution unit offers data access points for terminals, including radiofrequency capabilities and some signal processing functions. The network side focuses on functions such as data forwarding, traffic optimization, and content services. Utilizing distributed anchor points and flexible forwarding path settings, data packets are directed to the appropriate processing nodes to achieve efficient forwarding and comprehensive data processing, including deep packet inspection, content billing, and traffic compression.

#### Network Control Layer

This layer allows the 5G SA medical network to restructure and modularize its control functions. The primary function modules include centralized allocation of wireless resources; unified management and control of multiple access points; traffic grooming; and management of mobility, sessions, and security. According to the instructions provided by the 5G network function view of the management arrangement layer, the aforementioned functional components are integrated within the network control layer, allowing for flexible scheduling of resources.

#### Intelligent Operation Layer

It consists of 4 functional components: business data, user data, management arrangement, and network capacity opening. The business data function records information related to business processes and objects, and traffic statistics. The user data function stores information concerning user subscriptions, business policies, and network status. The management arrangement function utilizes network function virtualization technology to enable dynamic orchestration of network functions and the on-demand creation of network slices. The capacity opening function facilitates the centralized collection and encapsulation of network information, while also providing third parties access through application programming interfaces (APIs).

### Network Slicing Technologies

#### Advanced Technologies in 5G SA Medical Networks

**Figure 3:**
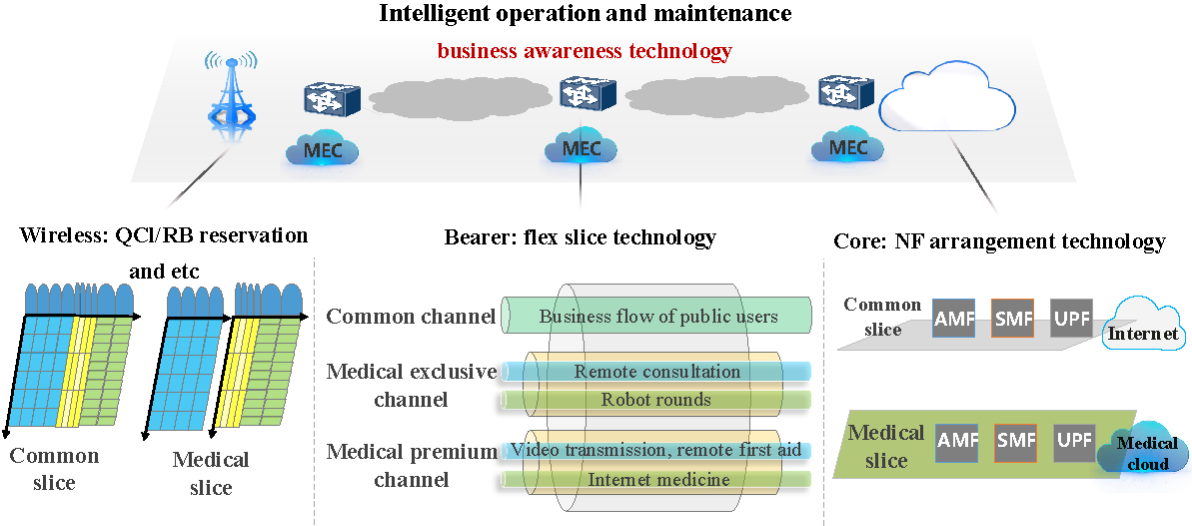
Technical architecture of the 5G SA medical private network. 5G: 5th generation mobile communication technology; AMF: access and mobility management function; NF: network element function; QCI: QoS class identifier; QoS: quality of service; RB: resource block; SMF: session management function; UPF: user plane function.

To meet the needs of various medical institutions, doctors, patients, and other stakeholders involved in medical services, advanced technologies (Table 1) such as network slice integration, edge cloud collaboration, and business awareness are used to construct the 5G SA medical network. This approach facilitates innovative applications such as on-demand deployment, high-speed connections, intelligent computing, and data security (Figure 3).

**Table 1:** Advanced technologies used to construct the private 5G^a^ SA^b^ medical network.

Technologies	Description
Network slice	The key feature of network function virtualization applied in 5G, which involves slice selection and management. Slice selection establishes access mapping between the user terminal and the network slice. Slice management also provides a safe isolation and highly automatic dedicated logical network for different medical services, hospital departments, and doctors.
Edge cloud collaboration technology	A technology used to achieve business linkage and resource sharing between edge computing and medical cloud systems; also used to realize the collaboration between resources, data, and applications.
Service-aware technology	A technology used to achieve the security, visualization, control, and management of the medical network and services.

^a^5G: 5th generation mobile communication technology.

^b^SA: standalone.

#### Access Network Slicing Technology

**Figure 4:**
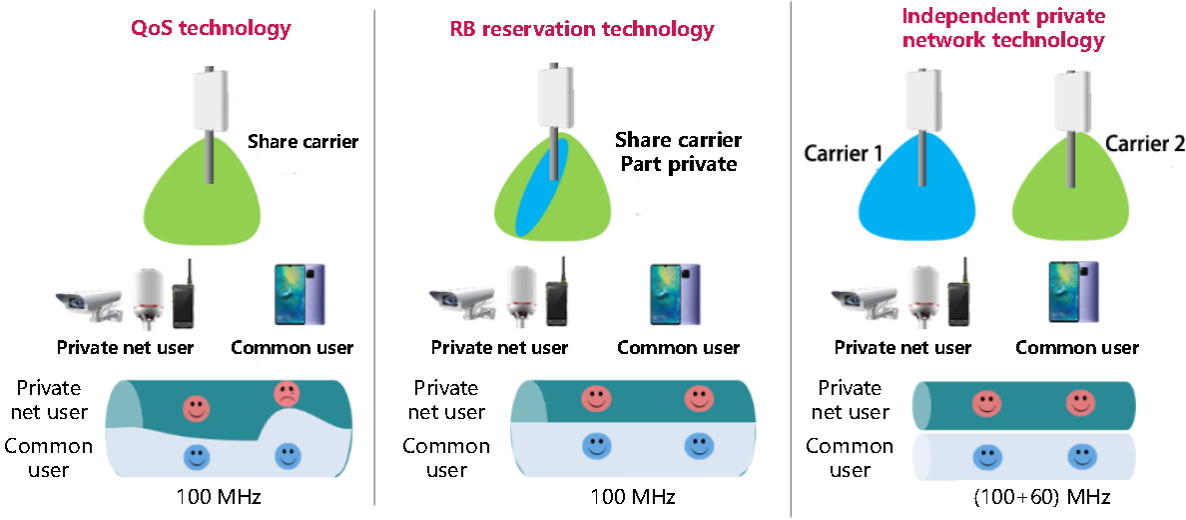
Three special network slicing techniques for the 5G base station access. 5G: 5th generation mobile communication technology; QoS: quality of service; RB: resource block.

The 5G base station access network, as a crucial component of the medical system, can utilize 3 specialized network slicing technologies to meet layering and grading requirements: quality of service (QoS) technology, resource block (RB) reservation technology, and independent private network technology (Figure 4).

QoS technology primarily allows mobile phone users, as well as small medical institutions and clinics, to access network services anytime and anywhere. For ordinary user terminal business, the access point name uses an ordinary priority default bearer, and the QoS class identifier (QCI) is set to 9, with the SIM card account selecting the QCI. For the dedicated mobile terminals of doctors, an access point name is used to establish a special bearer on demand, with the QCI set to 3. QoS is configured through control program facilities or policy and charging rule functions based on the terminal access controller. This QoS technology prioritizes services for doctor-specific mobile terminals, thereby enhancing the end-user experience when accessing the medical private network.

RB reservation technology is primarily designed to provide network services with business security for small and medium-sized medical institutions. RBs serve as the scheduling resources on the 5G access network side, and all services must request RB resources to be forwarded. This technique involves reserving RB resources and binding slice IDs based on medical service types and industry users, ensuring that only medical services with matching slice IDs can access the reserved RB resources. This approach enhances the security of medical service data and improves QoS guarantee capabilities.

Independent private network technology primarily offers exclusive network services for large medical institutions. Public and private network base stations can be deployed in the same physical area to ensure complete physical isolation. This technology utilizes the industry-specific 4.9 GHz band, while ordinary users operate on the 2.6 GHz band.

#### Bearing Network Slicing Technology

**Figure 5:**
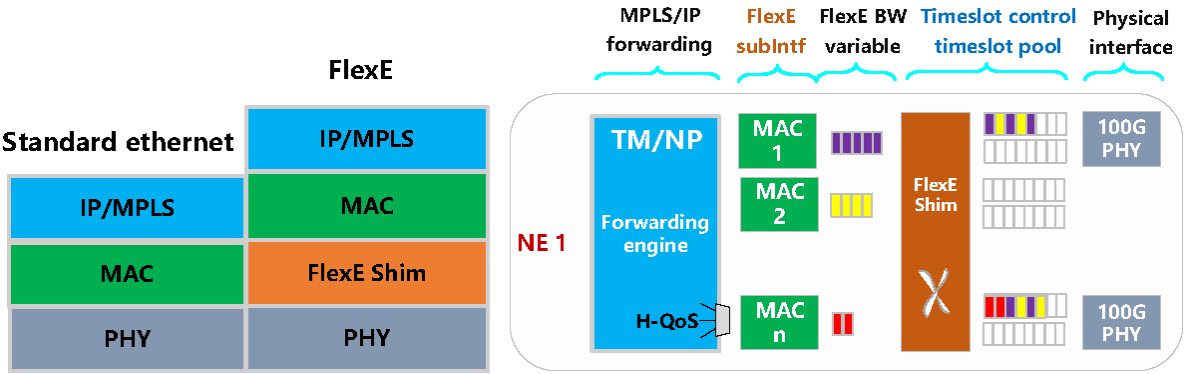
The FlexE Shim layer. FlexE: Flexible Ethernet; IP: internet protocol; MAC: media access control; MPLS: multiprotocol label switching; NP: network processor; PHY: physical layer; QoS: quality of service; TM: traffic management.

As illustrated in Figure 5, the 5G bearer network slicing primarily uses the international standard FlexE technology, introducing a FlexE Shim layer to achieve decoupling of the media access control (MAC) and physical layers. Additionally, it utilizes the FlexE time slot multiplexing technology to divide the physical port of the operator’s bearer network into multiple subchannel ports. This allows for complete isolation of services between each slice at the forwarding level through hardware time slot multiplexing, offering superior isolation compared with other forwarding isolation technologies. The 5G SA medical network slice, built on the original FlexE technology, uses time slot separation technology to enhance the granularity of the 5 Gbps slice to a more refined 1 Gbps granularity for medical-specific network slices. This solution offers an expanded virtual slice network for medical services, enabling efficient management and support of diverse medical business scenarios while simultaneously reducing the costs associated with constructing a private medical network.

#### Core Network Slicing Technology

The operation of core network subslicing relies on the collaborative functioning of various network element functions (NFs), including logical function entities defined by the 3GPP standards, such as the access and mobility management function, session management function, user plane function (UPF), unified data management, and policy control function [20]. The 5GC network supports a flexible combination of standard NF services alongside common services defined by the 3GPP. The visual interface enables the flexible arrangement of NFs through a drag-and-drop feature, facilitating the creation of customized network slices. Core network slice isolation technology primarily refers to the combination of varying degrees of isolation for each NF within the slice, allowing each NF to select its level of isolation based on the specific application scenario.

The NF isolation in the core network primarily adopts the following methods based on the requirements of service-level agreements (SLAs), cost considerations, and security isolation. First, the partial control method utilizes a specific user interface, offering moderate safety isolation while supporting multiple slices for the same terminal. This approach balances general costs with ease of deployment. Second, the specific user interface method focuses on isolating particular scenarios, which has the lowest cost but can be challenging to deploy. Currently, the medical field is well-suited for user-specific solutions. Lastly, the full isolation method separates the control interface from the user interface, making it suitable for scenarios with high-security isolation or customization demands. However, this approach entails the highest cost and moderate deployment difficulty. To address the diverse needs of various smart medical applications, the core network slice isolation adopts a dedicated scheme for the user plane. The UPF is deployed separately for different industries, relying on open and reliable connections, as well as computing and storage resources, to support the flexible delivery of multiecological services at the access edge.

#### Edge Cloud Collaboration Technology

Edge cloud collaboration targets different levels of medical institutions and business-to-business medical application scenarios. It relies on provincial-, municipal-, and county-level medical facilities to establish a distributed medical service system, including stationed edge clouds and prefectural and municipal medical clouds. This approach fully leverages edge computing capabilities, such as real-time or accelerated data processing and analysis, efficient network traffic management, and enhanced local data security. The distributed medical service system facilitates collaboration among multilevel medical clouds, edge networks, resources, and services. Specifically, the provincial medical cloud primarily serves large medical institutions at the provincial level, deploying industry-level collaborative and massive computing operations. It acts as the core for business release, management, and supervision, overseeing collaboration across all edge networks. The municipal center cloud functions as a city-level medical collaboration center, managing the business of the provincial medical cloud and overseeing the release, management, and supervision of city-level medical operations. Coordinating with the provincial medical cloud, the municipal center cloud oversees the management of all stationed edge clouds within the city. The stationed edge cloud primarily supports the business endpoints and data storage centers of county-level medical institutions, enhancing the response efficiency of real-time operations by diverting local business and implementing real-time processing. For collaborative tasks or those requiring significant computational power, data are uploaded to the city-level central cloud for calculation and processing.

#### Service Aware Technology

#### Medical Network Management and Control Platform

It is imperative to establish a medical network management and control platform to support diverse medical services. Leveraging advanced networking technologies, including visualization, manageability, control, and security monitoring, the platform can create a visual, manageable, and controllable environment for medical networks, business operations, and security.

#### Network Visualization

By utilizing the millisecond-following flow detection technique of in-band flow information telemetry (IFIT), alongside telemetry reporting and intelligent analysis from the network management system, network visualization can monitor service SLA in real time. This capability allows for quick fault delimitation and localization, enabling rapid recovery of services within minutes. SLA visualization technology can be effectively applied in the operation and maintenance of large-scale medical private networks, significantly enhancing efficiency.

#### Network Coordination

Based on the Route and Area Matching (ROAM) intelligent routing algorithm, in conjunction with the original routing algorithm, the system selects the optimal path according to various network requirements, including bandwidth and service time delay. This approach addresses the limitations of traditional routing algorithms, which typically focus solely on the shortest path. By enabling path planning and dynamic optimization at the service level, it enhances the overall utilization rate of the medical network.

#### Network Control

The management and control platform monitors slice bandwidth utilization and historical service concurrency in real time, allowing for reasonable planning of slice sizes. In the event of network congestion, the platform automatically detects the utilization rate of network slices and dynamically adjusts their bandwidth to ensure the smooth operation of medical services.

#### Security Detection

Security in 5G medical networks encompasses the protection of terminal access, network transmission, and system applications. Terminal access security ensures that 5G terminals utilize a special network card and implement security measures through native protocols, such as the 5G Authentication and Key Agreement mechanism, as well as 2-way authentication, to safeguard access to the network. To ensure visibility, manageability, and controllability of network security in the medical industry, security measures such as deploying security equipment or software—including the UPF security gateway, NF isolation, IFIT, telemetry, firewalls, and advanced persistent threat intrusion detection—are implemented. Additionally, system application security focuses on delivering network and business security services to users within the medical sector. To ensure the security of terminals, data, and systems in medical businesses, various security mechanisms are used, including system log-in verification, face and fingerprint recognition, certification authority signature authentication, management of system permissions, and data desensitization for medical information sharing.

### Ethical Considerations

Ethical approval of this study was granted by the Ethics Committee of the First Affiliated Hospital of Zhengzhou University (No.2020-KY-381).

## Results

### Construction of a Private 5G SA Medical Network

#### Framework Overview

**Figure 6:**
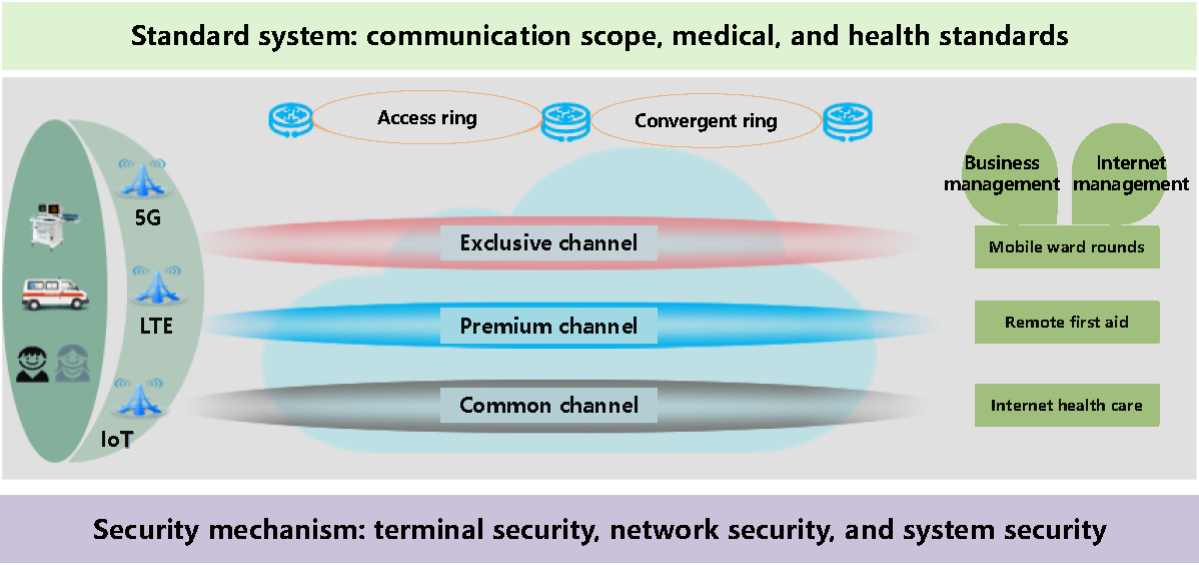
The proposed 5G SA medical network in a smart health environment. 5G: 5th generation mobile communication technology; IoT: Internet of Things; LTE: Long Term Evolution; SA: standalone.

The private 5G SA medical network is constructed on key 5G technologies and tailored to meet the specific needs of medical services. This innovative network offers a range of services, including bandwidth, latency, connection capacity, and security isolation, addressing diverse network requirements. It utilizes various slicing technologies in the wireless access, transport, and core networks. This facilitates the transformation of traditional network architecture, allowing for the selection and management of medical slices tailored to different users in health care settings. This ensures prompt responses to various medical services while promoting the safe, standardized, and efficient operation of health care operations. The proposed 5G SA medical network within a smart health environment is illustrated in Figure 6. The construction of the 5G network primarily encompasses the medical application system, information communication standards, and network security mechanisms.

As depicted in Figure 6, the private 5G SA medical network offers customized virtual network slicing services categorized into 3 types: exclusive, premium, and common networks. This structure meets the high-reliability network requirements for various application scenarios, including mobile ward rounds, remote first aid, and internet health care, while also optimizing the user’s network experience. The medical exclusive channel supports services accessed by fixed service sites of medical institutions and allows connectivity to various networks, including 3G/4G Long Term Evolution (LTE), Narrowband IoT, and Wi-Fi. The premium channel is designated for out-of-hospital mobile terminal access scenarios. In cities and counties that support these mobile services, a dedicated network virtual slice is preallocated for the access ring in the region, with real-time synchronization of the routing tables across all access rings in the entire network. During actual business operations, multiple subnetwork slices are connected to the same premium channel to ensure that out-of-hospital mobile business data remain within the hospital, thereby protecting patient privacy and information security. After allocating bandwidth to the exclusive and premium channels, the remaining bandwidth is automatically assigned to the common channel, with network bandwidth resources dynamically adjusted based on actual business needs.

#### Deployment Framework

Through end-to-end network slicing technology, the 5G SA medical network allocates independent resources across the “province-city-county” hierarchy, ensuring rapid and comprehensive coverage of medical institutions at all levels. This approach achieves isolation between medical and public services, safeguarding medical operations from any potential impact caused by public services. The 5G SA medical network supports access through 5G, 4G, Wi-Fi, the internet, and dedicated virtual private network lines. The iGW-S gateway is deployed in the hospital’s machine room to facilitate access to fixed devices and edge cloud services. In large- and medium-sized hospitals or operator data centers, the iGW-M and UPF are utilized to manage the diversion of private network services. Small hospitals can lease the operator’s iGW-C gateway, which supports access for mobile devices within the hospital and for vehicle-mounted devices outside of it. Mobile and internet medical users access the regional medical cloud through the operator’s iGW-C, significantly expanding the coverage of smart medical services. Medical alliances and communities connect to the iGW-M via either the iGW-C or iGW-S to access the edge or medical cloud. The iGW-M then uniformly distributes interhospital collaborative traffic to the edge or medical cloud, facilitating business collaboration between fixed and mobile access units. This centralized access point ensures network connectivity across the entire system, simplifies network connections at the business layer, and guarantees the efficient and high-quality execution of medical services.

#### Physical Components of the Hardware System

To implement the 5G SA network, various hardware components are utilized, including 5G base stations, 5GC networks, transmission networks, edge computing systems, antennas, power and environmental monitoring devices, network management tools, and user terminals. The main physical components are outlined in Table 2. Together, these hardware devices form the infrastructure of the 5G SA network, enabling the delivery of high-bandwidth, low-latency, and highly reliable communication services.

**Table 2:** The main physical components of hardware used in the 5G^a^ SA^b^ network.

Hardware	Physical components	Description
5G base station equipment	Antenna, radiofrequency module, baseband processing unit	Wireless access points of the 5G network and communicating with user terminals
5G Core network equipment	Active antenna unit, distributed and centralized unit, access and aggregation interface, user plane function, session management, and access and mobility management	Supporting key 5G features such as high-speed data transmission, low-latency communications, and large-scale device connectivity
Transmission network	Optical transmission equipment, IP/MPLS^c^ routers, and switches	Connecting the 5G base stations and core network
Edge computing equipment	Smart cameras, Internet of Things gateways, edge servers, and micro data centers	To handle delay-sensitive services and reduce the burden on the core network
Antennas equipment	Massive MIMO^d^ antenna system	Improving network capacity and coverage
Equipment of power and environmental monitoring	Two-circuit mains power supply or UPS^e^ power supply, and intelligent power detector	Providing power support for base stations and core networks, and monitoring the operating status of hardware
Network management equipment	Software-defined networking controller, fiber optic switches, and virtual private network gateways	Managing and monitoring the entire 5G SA network
User terminals	Mobile phones, 5G portable Wi-Fi, customer terminal equipment, and Internet of Things	Communicate directly with the 5G base stations and support the 5G SA network

^a^5G: 5th generation mobile communication technology.

^b^SA: standalone.

^c^IP/MPLS: Internet Protocol/Multi-Protocol Label Switching.

^d^MIMO: Multiple Input Multiple Output.

^e^UPS: uninterruptible power supply.

#### Medical Application System

The 5G SA network is compatible with existing network modes, including 3G, 4G LTE, and IoT. To address the diverse network requirements of medical services, it utilizes network slicing and virtualization technology to construct exclusive, preferential, and common channels. This approach supports medical business operations and network management, facilitating seamless integration of mobile ward rounds, remote first aid, and internet-based health care services across intrahospital, interhospital, and out-of-hospital scenarios (Figure 6).

#### Information Communication Standards

The network adheres to the relevant standards of 3GPP R15 for wireless and transmission networks, as well as core network slices. It provides precise definitions for slices, message queuing telemetry transport protocol, differentiated scheduling processing, and resource mapping, ensuring the standardization of network construction. Additionally, it supports Health Level Seven (HL7), the 10th revision of the International Classification of Diseases (ICD-10), and other medical and health information communication standards, facilitating the interaction and sharing of various medical service information.

#### Network Security Mechanism

The security of the 5G SA network is underpinned by technologies such as business slice isolation, software-defined networking, certificate authority authentication, hierarchical decentralization, and blockchain. These technologies effectively manage terminal access and identity while ensuring secure data access and application processing. The 5G network provides security authentication, abnormal behavior analysis, and dynamic access control for medical terminals, networks, and application systems, thereby safeguarding the integrity of the medical private network.

### Applications of the Private 5G SA Medical Network

#### Robot-Assisted Mobile Ward Round

**Figure 7:**
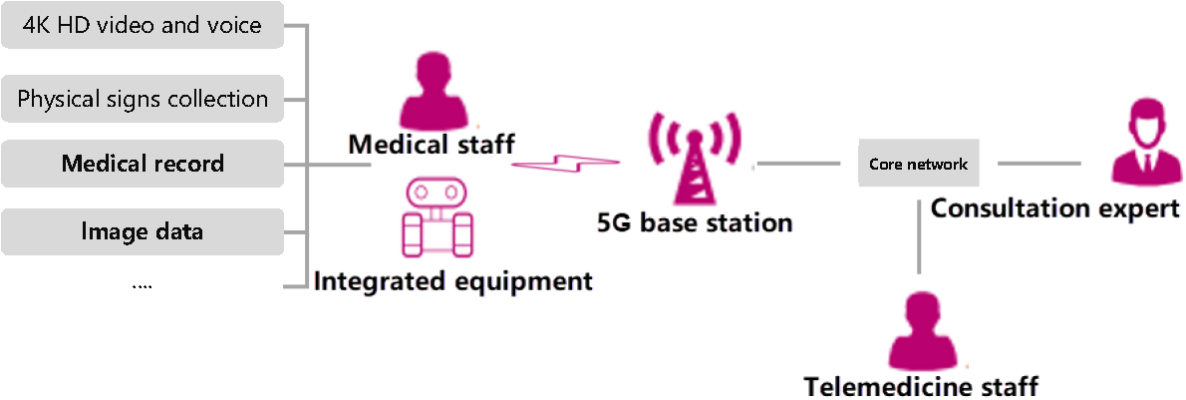
The 5G-enabled robot-assisted mobile ward rounds. 5G: 5th generation mobile communication technology.

Robot-assisted mobile ward rounds extend medical care services directly to the patient’s bedside. This system is built on the IoT, edge computing, data fusion, and robotics, integrating various medical information systems within hospitals. It enhances daily nursing ward rounds by enabling medical professionals to utilize the 5G network to access medical data acquisition terminals, audio and video interaction systems, electronic medical records, and robot control systems. This integration supports efficient medical data transmission and nondestructive data interaction. Consequently, the homogenization of mobile ward round business services can be achieved, ultimately enhancing the quality and efficiency of ward rounds and nursing services (Figure 7). Additionally, in specialized areas such as isolation wards and environments with electromagnetic radiation, health care professionals can maneuver medical service robots to designated beds for conducting remote ward rounds and providing nursing services. This further ensures the personal safety of medical staff.

The robot-assisted mobile ward rounds primarily encompass application scenarios such as IoT-based data collection, remote control of clinical examinations and tests, and audio-video interaction for telemedicine. To meet the actual needs of medical staff for clinical diagnosis and treatment—including real-time control of smart terminals, stable and reliable mobile terminals, and secure data exchange—it is both necessary and feasible to establish a 5G SA medical private network. This network will provide differentiated and secure support for medical services. As shown in Table 3, the performance of 4G is inadequate to meet the transmission requirements for 4K high-definition video, control information, and medical data. Regarding terminal mobility, under conditions of network security and stability, the actual terminal mobile speed for 4G is 200 km/h, which is 250 km/h lower than the 450 km/h achievable by 5G terminals. Additionally, 4G lacks dedicated transmission channels for industry users, making it challenging to ensure adequate security. The proposed 5G SA network supports network slicing, allowing for the allocation of multiple slices specifically for smart medical networks. It provides dedicated wireless air interface resources and control management for medical services. This ensures the independence and isolation of these services, thereby enhancing the feasibility, real-time capabilities, reliability, and security of medical services.

During the COVID-19 epidemic, medical staff could remotely control a mobile ward inspection robot to enter the isolation ward via an exclusive 5G medical service channel. This enabled them to gather vital signs, such as body temperature and electrocardiograms, from patients, as well as access electronic medical records. The system also facilitated collaboration with external experts for multidisciplinary consultations and provided remote guidance to medical staff in the isolation ward for patient rescue. The medical exclusive channel creates a clear separation between medical services and public affairs, mitigating the impact of sudden influxes of public activity during critical periods. It establishes a seamless connection between health care professionals and patients. To conduct network testing and service applications during the COVID-19 epidemic, mobile ward rounds utilizing the proposed 5G SA medical network were implemented in the isolation ward area of the First Affiliated Hospital of Zhengzhou University. The field test results showed a peak 5G download rate of 918 Mbps and an average download rate of 790 Mbps. The peak upload rate was 94.15 Mbps, with an average upload rate of 91 Mbps.

**Table 3:** The feasibility of the 5G^a^-enabled robot-assisted mobile ward rounds.

Items and requirements of typical applications			Indicators		Basic needs		4G^b^		5G SA^c^
**Expert side**									
	High-definition lossless transmission of doctor’s 4K video	Downlink		36 Mbps		20 Mbps		91 Mbps	
	No-sense real-time transmission of control information	Latency		10 ms		20-30 ms		1 ms	
	Medical data	Downlink		200 Mbps		100 Mbps		790 Mbps	
**Robot side**									
	High-definition lossless transmission of patient’s 4K video	Uplink		36 Mbps		20 Mbps		91 Mbps	
	No-sense real-time transmission of control information	Latency		10 ms		20-30 ms		1 ms	
	Medical data	Uplink		50 Mbps		20 Mbps		91 Mbps	
**Mobile terminal**									
	Stable and reliable network	Speed		300 km/h		200 km/h		450 km/h	
**Security**									
	Security interaction of medical privacy data	Data security		Data security access		Network reuse and no security guarantee		FlexE^d^ logical slicing, hard isolation, and high security	

^a^5G: 5th generation mobile communication technology.

^b^4G: 4th generation mobile communication technology.

^c^SA: standalone.

^d^FlexE: Flexible Ethernet.

#### Remote Prehospital First Aid

Prehospital first aid is a crucial component of emergency medicine, aimed at improving the rescue efficiency of patients before they reach the hospital, as well as reducing disability and mortality rates. By implementing the 5G SA medical network, the digital prehospital first aid system at the emergency center can equip first aid vehicles with essential medical devices such as monitors, electrocardiographs, emergency breathing machines, defibrillation monitors, and cardiopulmonary resuscitators. By adopting technologies such as mobile communication, IoT, and audio and video communication, and centering on the prehospital first aid electronic medical record system, we can integrate multidimensional systems for vital signs monitoring, mobile vehicles, vehicle navigation, GPS positioning, and emergency command and dispatch. With this design and construction, remote prehospital first aid can support the transmission of video images and inspection results to emergency command centers and hospitals through 5G premium channels, whether ambulances are stationary or in motion. It also enables real-time transmission of information related to medical equipment monitoring, vehicle positioning, and video footage both inside and outside the vehicle. Furthermore, it facilitates the collection, processing, storage, transmission, and sharing of information pertinent to remote prehospital first aid. If needed, clinical experts can be connected through the prehospital emergency system for remote rescue guidance.

**Figure 8:**
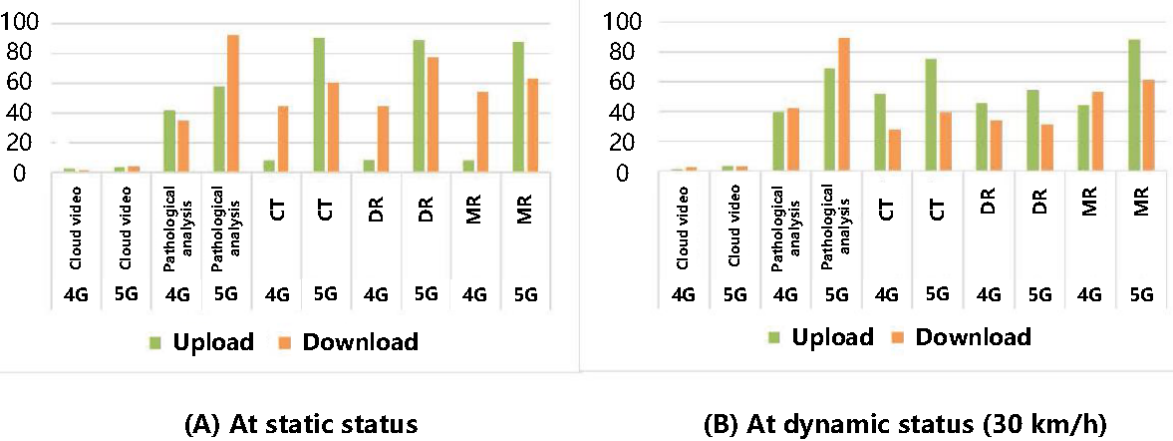
Comparison of uplink and downlink average service bandwidth of the 4G/5G-enabled remote first aid service at static/dynamic status. 4G: 4th generation mobile communication technology; 5G: 5th generation mobile communication technology; CT: computed tomography; DR: digital radiography; MR: magnetic resonance.

To verify the effectiveness of the 5G SA network in the prehospital emergency system, the First Affiliated Hospital of Zhengzhou University established a network test environment in the Longzihu area of Zhengzhou City. They conducted both static and dynamic service tests on ambulances equipped with the remote first aid system. The network bandwidth, jitter, delay, and other performance parameters of the system were recorded during operation under various conditions. These data were then used to compare and analyze network changes during actual application. The test results demonstrated that the 5G SA network effectively meets the requirements of diverse business applications in prehospital emergency scenarios (Figure 8). The upstream bandwidth of 4G was clearly insufficient compared with 5G, exhibiting poor delay stability and a significant range of fluctuations, especially during regional boundary switches. Additionally, in high-load networks with concurrent multiservice usage, the performance of the 4G network deteriorated more rapidly than that of 5G.

#### Remote Ultrasound

The noninvasive nature of medical ultrasonography greatly contributes to its widespread clinical use, which largely depends on the scanning techniques employed by the physician. Selecting an incorrect scanning area can lead to misdiagnosis and inappropriate treatment of patients. Remote ultrasound experts can leverage the low-latency 5G network to control the robot arm in real time using a handheld simulation ultrasound device. This capability enables them to perform ultrasound examinations for patients in county-level hospitals and provide diagnostic recommendations to local doctors. The full automation of real-time remote ultrasound diagnosis across regions and hospitals minimizes the medical and nursing operations required at the patient’s side. As a result, the quality of diagnosis and treatment, as well as the level of ultrasound imaging services in primary hospitals, can be significantly improved.

**Figure 9:**
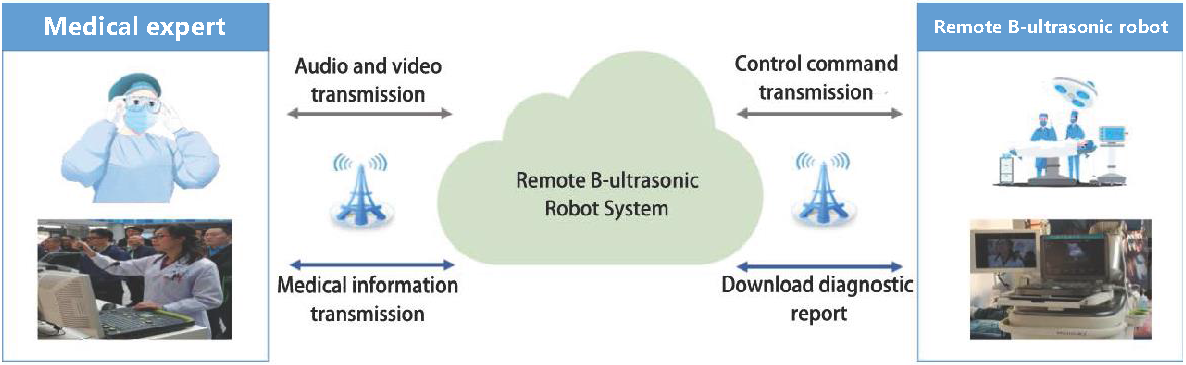
The 5G-enabled remote ultrasonic examination. 5G: 5th generation mobile communication technology.

Compared with 4G, the latest 5G technology offers a transmission rate that is 20 times higher, mobile speeds that are 1.5 times faster, and latency that is 10 times lower. In addition to its high bandwidth, low latency, and extensive connectivity, 5G can support multichannel transmissions of high-definition video and precise ultrasound images. It also enables the simultaneous networking of a significant number of medical devices both inside and outside of hospitals. Thus, the quality of images transmitted and received by the 5G-powered remote ultrasound diagnostic system surpasses the limitations of the 4G network and meets the requirements for remote ultrasound services. In this study, to verify the feasibility of remote ultrasound, a 5G-enabled ultrasound examination system was implemented between the First Affiliated Hospital of Zhengzhou University and Luoning County People’s Hospital (Figure 9). The test results indicated that the average downlink rate of the 5G network was 4.82 Mbps, while the average uplink rate reached 2 Mbps, with an average fluctuation of approximately 8 ms. The system delay of 83 ms was significantly lower than the perceptible artificial delay of 200 ms, thereby meeting the practical requirements for remote ultrasound examinations.

### Performance Tests

Given the specific requirements of medical applications in intrahospital, interhospital, and out-of-hospital scenarios, this study conducted static and dynamic business tests under both 4G and 5G conditions to evaluate the performance of the proposed 5G SA medical private network. The tools used during the performance tests are listed in Table 4. The testing environment for the 5G network includes a 5G macro station, 64T64R, customer premise equipment (CPE) access, a core network SA, and bearer network packet transport network equipment. By contrast, the 4G environment comprises a 4G macro station, 8T8R, mobile phone or CPE access, a core network EPC, and bearer network packet transport network equipment. The tests record performance parameters such as network bandwidth, jitter, and latency during system operation under different conditions. These data are then used to compare and analyze network changes during the actual use of medical services under both 4G and 5G conditions, aiming to support the implementation of 5G smart medical applications.

**Table 4:** Details of the testing tools.

Testing tools	Description
Flow instrument (Sunrise Telecom)	Simulates network congestion
5G^a^ New Radio CPE^b^	5G network switches to wired broadband
HUAWEI mate 20 X (5G packet filling test)	Mobile testing
HUAWEI mate 20 X (4G^c^ card)	Pathology, consultation, and electrocardiogram tests
4G CPE	Cloud video testing in a 4G environment
GENEX Probe 5.2 (testing software; Informer Technologies, Inc.)	Provides upload/download/ping services for both server side and client side

^a^5G: 5th generation mobile communication technology.

^b^CPE: customer premise equipment.

^c^4G: 4th generation mobile communication technology.

**Figure 10:**
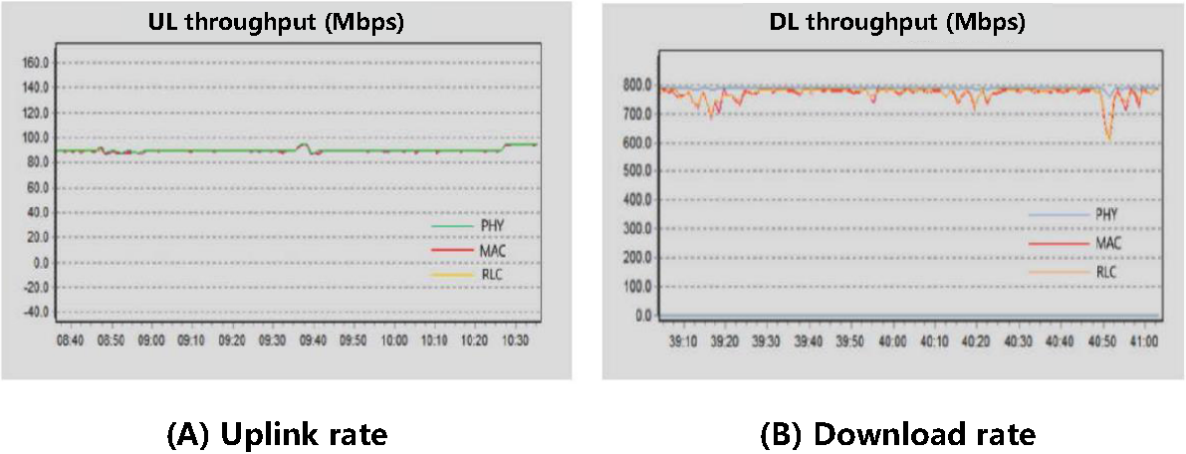
Bandwidth test of the private 5G SA medical network. 5G: 5th generation mobile communication technology; DL: download; MAC: media access control; PHY: physical layer; RLC: radio link control; SA: standalone; UL: uplink.

The 2R2T CPE at the test site of the First Affiliated Hospital of Zhengzhou University is connected to the 4.9G macro station and accesses the SA core network via an exclusive network channel. The doctor’s workstation connects to the CPE using a standard 6-network cable, while traffic and delay changes through the CPE are monitored using GENEX Probe software. During the bandwidth testing process, data packets were continuously sent to the core network server using user datagram protocol (UDP), with the CPE connected to the nearest base station for relevant testing. The measured uplink rate of the 5G SA medical network, as shown in Figure 10A, remained consistently stable at 90 Mbps. The download rate of the network is depicted in Figure 10B, with the physical layer’s rate remaining stable at 800 Mbps. The MAC layer and the radio link control layer are components of the data link layer. The compression and encryption processes that occur when information transitions from the physical layer to the data link layer can lead to attenuation and fluctuations in the download rate, which is considered a normal occurrence.

**Figure 11:**
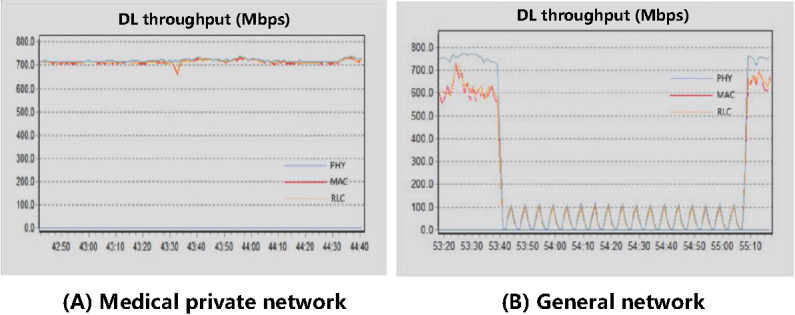
Comparison between the medical private network and the common network before and after the flow. DL: download; MAC: media access control; PHY: physical layer; RLC: radio link control.

The background flow is created using a flow meter, which occupies the physical pipeline to simulate a congested scenario. The 5G medical network slice has a bandwidth of 1 Gbps, while the ordinary user slice offers a bandwidth of 3 Gbps. The background traffic measures 2.935 Gbps, with the pulse signal entering the ordinary user slice. The medical special network service, ordinary user service, and background flow service share the network port. The test results are shown in Figure 11. Before the background stream was introduced, the downlink rate of the medical private network remained stable at over 700 Mbps, while the download bandwidth of the ordinary user service fluctuated around 600 Mbps. After the background stream was input, the download bandwidth of the 5G medical network service remained unaffected, whereas the download rate of the ordinary user service dropped to below 100 Mbps.

Before and after the network congestion, ordinary user services were significantly impacted, as background stream activities consumed a substantial portion of the bandwidth allocated for normal operations. This resulted in a significant decrease in download speeds for regular users. By contrast, the user services of the medical private network remained largely unaffected after the congestion, fully meeting the demands for high concurrency in medical services.

**Figure 12:**
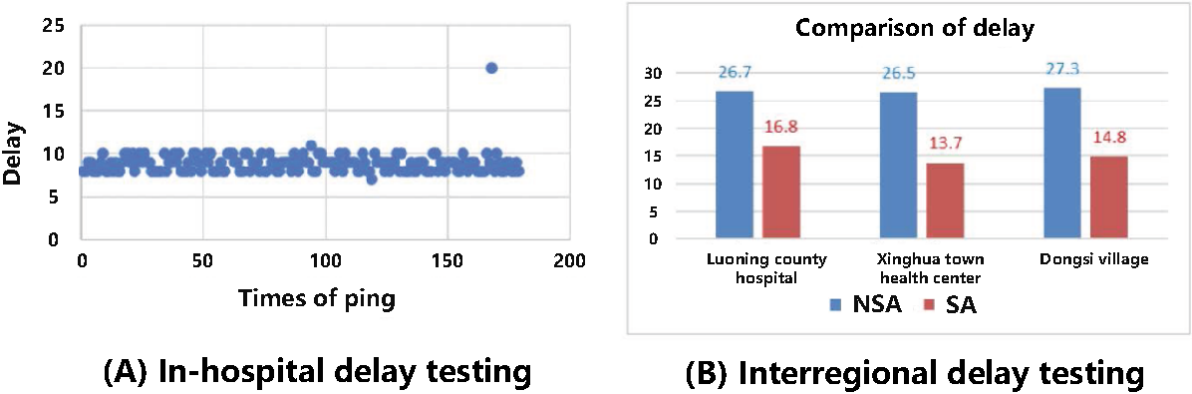
Delay test of the private 5G SA medical network. 5G: 5th generation mobile communication technology; NSA: non-standalone; SA: standalone.

Interhospital cross-regional services, such as remote ultrasound, require stricter time delay standards. This study established a test environment to conduct delay jitter tests both within and between hospitals. During in-hospital testing, ping packets were sent directly to the doctor at the remote ultrasound workstation, using a batch size of 1000 messages, each 32 bytes in length. In 180 Ping tests, 178 results fell between 8 and 10 ms, with an average delay of 8.86 ms. During the interhospital tests, Luoning County Hospital, Xinghua Town Health Center, and Dongsi Village were all directly connected to the local 64T64R 5G macro station via 2R2T CPE. Additionally, the doctor workstation at the First Affiliated Hospital of Zhengzhou University sent Ping packets to each county hospital’s workstation. The test results are presented in Figure 12. Compared with the NSA network, the SA network demonstrated a significant reduction in delay, with the average interhospital latency measured at 14 ms.

## Discussion

### Principal Findings

Based on a comprehensive requirements analysis and the design of the system and functional architecture, this study developed and launched a private 5G SA medical network using key technologies such as end-to-end elastic network slicing, edge cloud collaboration, and service awareness. The construction and functionality of the network were explored, along with 3 typical applications and performance tests of the proposed 5G SA medical network. To our knowledge, this is the first comprehensive account of a private 5G SA medical network explored in a smart health environment based on real-world practice in China. The findings can serve as a valuable reference for the further development and implementation of 5G medical networks in China and other regions worldwide.

The characteristics and advantages of 5G are precisely what current medical services urgently require. Challenges that remained unresolved during the 4G era—such as the interconnection of intrahospital equipment, the implementation of interhospital medical services, and the effective integration of out-of-hospital emergency treatment with regional health care systems—can be swiftly addressed in the 5G era [5,6,12,21]. The advent of 5G facilitates the interconnection of all things, empowers mobile health care, enhances AI performance, and accelerates the industrialization of smart medical care [22]. Compared with NSA, SA offers 2 distinct advantages. First, network slicing in SA supports differentiated SLAs for medical services, providing varying QoS guarantees for medical applications. This improves network service quality and reduces deployment costs [23,24]. Second, SA facilitates the implementation of distributed cloud data centers. The mobile edge computing platform on the hospital side allows users to connect directly with the edge node, enhancing the efficiency and effectiveness of processes and operations. With extremely low network latency, SA enables applications in medical service robots, remote surgery, and other medical services [25,26]. Although the deployment costs for SA are higher than those for NSA, its operational expenses are comparatively lower. Therefore, while NSA may serve as an interim solution, it is expected to be replaced by SA in the future [9].

In terms of the proposed system design in this study, the advantages of a private 5G SA medical network with cloud-native characteristics are primarily reflected in the following aspects [27,28]. First, network compatibility: building on traditional medical networks, it supports multiple access modes such as satellite, Wi-Fi, wired networks, and cellular data networks. This versatility facilitates large-scale medical data collection and sharing, as well as collaboration in medical services. Second, the network slicing design utilizes different slicing technologies based on the functional positioning, business scenarios, and 5G characteristics of medical institutions. This allows for the hierarchical deployment of 5G networks and facilitates the flexible combination of medical private networks through decoupled microservices. As a result, diverse network requirements for various medical service scenarios can be promptly addressed. The isolation of medical services from public user services enhances the security of medical operations. Third, edge cloud collaboration involves deploying edge cloud systems at various levels of medical institutions. Real-time interactive tasks are offloaded to the edge cloud for processing, while non–real-time and computationally intensive tasks are uploaded to the medical cloud for execution. This approach improves the utilization of IT resources and enhances the real-time performance of medical services. Fourth, intelligent operation and maintenance leverage the types of medical businesses and historical data to analyze concurrent 5G network traffic, ensuring service-level commitments for bandwidth and latency. Minute-level fault location is achieved through the use of IFIT and telemetry, while medical application–level SLA guarantees are provided by implementing SLA slice selection and management functions.

The “Overall Plan for 5G Technology R&D and Testing” [29] released by IMT-2020 outlines clear performance index requirements. Specifically, the user experience rate should reach 100 Mbps in wide-area coverage scenarios and 1 Gbps in hotspot coverage areas, while the end-to-end delay must remain within 10 ms [30,31]. Based on the test results of the proposed 5G SA medical network, the current bandwidth, concurrency, and delay all meet the performance index requirements set by IMT-2020. Meanwhile, the logical bandwidth of the 5G SA medical network slicing at the First Affiliated Hospital of Zhengzhou University is 1G, with enhanced granularity, representing only one-fifth of the previous network slicing. This means that the proposed solution for the 5G SA medical network allows for the allocation of additional network resources, thereby promoting the development and implementation of differentiated medical applications [10,24].

For the construction, application, and performance of the 5G SA medical network in a smart health environment, this study makes several notable contributions. First, the proposed 5G SA network integrates network slicing and edge computing technologies, which effectively address the requirements for high bandwidth, wide coverage, low latency, and visualization in medical applications. Second, this study introduces a security mechanism tailored for the SA medical network in 5G to protect user privacy. Third, 3 typical applications of the 5G SA medical network were explored: robot-assisted mobile ward rounds, remote ultrasonic examinations, and virtual reality–enabled remote emergencies. Lastly, the 5G SA medical network underwent a series of experimental tests to accurately assess its performance, providing valuable references through specific indicators.

Based on the test results, the quality of the 5G SA medical network slicing can be assured and remains unaffected by public business emergencies. However, due to current network capacity limitations, several challenges persist. First, the level of integration between the medical industry’s private network and medical business operations is currently suboptimal and requires further optimization. To date, the application of network slicing has only achieved end-to-end network slicing and facilitated the construction of the 5G SA network. However, the current integration with medical business operations is insufficient. A crucial focus of future research on medical network slicing should be the integration between medical business microservices and medical slice microservices [1,7]. Second, achieving efficient deployment of end-to-end network slices is essential for the practical implementation of medical private networks. The end-to-end slice manager facilitates the rapid creation, deployment, and deletion of network slices. Additionally, slice deployment and upper-level applications in the medical industry can be automatically connected through relevant APIs, expediting the instantiation process and enabling large-scale deployment of the 5G medical network [20,32]. Third, the medical industry currently lacks sufficient 5G-enabled application scenarios, highlighting the need to optimize business models. Current 5G medical network application scenarios are predominantly confined to laboratory testing due to insufficient support from the 5G industry chain, limited wireless coverage, and a singular 5G application mode [3,21,26]. This situation presents several notable issues, including limited diversity in business types, unclear business demands, and ambiguous business models [32,33]. The next step should involve investigating and verifying additional medical applications, such as remote surgery and virtual medical teaching, along with other relevant scenarios. It is essential to focus on researching the business traffic model of the medical network that integrates 5G and wired networks, while further expanding and optimizing the private 5G medical network [21,22,34].

### Conclusions

The 5G network is capable of meeting diverse requirements for intelligent medical equipment access, patient privacy security, the complexity of medical scenarios, and the mobility of medical services. In this study, a private 5G SA medical network was proposed for a smart health environment. On the one hand, the construction and application of the 5G SA medical network could give rise to a series of innovative medical service models. It provides a robust network foundation for the rapid development of new service models, including medical service robots, AI-assisted diagnosis, and virtual doctors. Simultaneously, the incubation of related industries will enhance the efficiency of medical services and improve the interactive experience for patients. On the other hand, the 5G SA medical network serves as a significant reference for the design and implementation of specialized networks in other industries. The objective of this study is to establish a differentiated 5G SA private medical network that caters to various levels of medical institutions and their corresponding services. This network not only meets the requirements for the development and implementation of medical services such as mobile medical care, telemedicine, and internet-based health care, but also offers construction ideas and reference plans for 5G deployment in other sectors, including agriculture, households, transportation, and power.

## Abbreviations

3GPP: 3rd Generation Partnership Project
4G: 4th generation mobile communication technology
5G: 5th generation mobile communication technology
5GC: 5G Core
AI: artificial intelligence
API: application programming interface
CPE: customer premise equipment
EPC: evolved packet core
FlexE: Flexible Ethernet
HL7: Health Level Seven
ICD-10: 10th revision of the International Classification of Diseases
IFIT: in-band flow information telemetry
IoT: Internet of Things
LTE: Long Term Evolution
MAC: media access control
NF: network element function
NSA: non-standalone
QCI: QoS class identifier
QoS: quality of service
RB: resource block
ROAM: Route and Area Matching
SA: standalone
SLA: service-level agreement
UPF: user plane function
